# Insulating Innovative Geopolymer Foams with Natural Fibers and Phase-Change Materials—A Review of Solutions and Research Results

**DOI:** 10.3390/ma17184503

**Published:** 2024-09-13

**Authors:** Agnieszka Przybek, Michał Łach

**Affiliations:** Faculty of Material Engineering and Physics, Cracow University of Technology, Jana Pawła II 37, 31-864 Cracow, Poland

**Keywords:** geopolymer foam, insulating innovative material, natural fiber, phase-change material (PCM), organic additive for CO_2_ reduction

## Abstract

Geopolymers are synthesized using anthropogenic raw materials and waste from the energy industry. Their preparation necessitates an alkaline activator, which facilitates the dissolution of raw materials and their subsequent binding. At present, geopolymers are considered a promising material with the potential to replace conventional cement-based products. This research investigates foamed geopolymer materials based on fly ash, natural fibers, and phase-change materials. The study utilized three distinct types of fibers and two phase-change materials manufactured by Rubitherm Technologies GmbH of Germany. This paper presents the results of the thermal conductivity coefficient and specific heat tests on the finished foams. Additionally, compressive strength tests were conducted on the samples after 28 days. Natural fibers decreased the insulation parameter by 12%, while PCM enhanced it by up to 6%. The addition of fibers increased the compressive strength by nearly 30%, whereas PCM reduced this by as little as 14%. Natural fibers and phase-change materials had an increased heat capacity by up to 35%. The results demonstrated the material’s potential in various industrial sectors, with the primary areas of application being building materials and insulations. The findings illustrate the significant potential of these composites as energetically and environmentally sustainable materials.

## 1. Introduction

### 1.1. Problem Analysis

**Figure 1:**
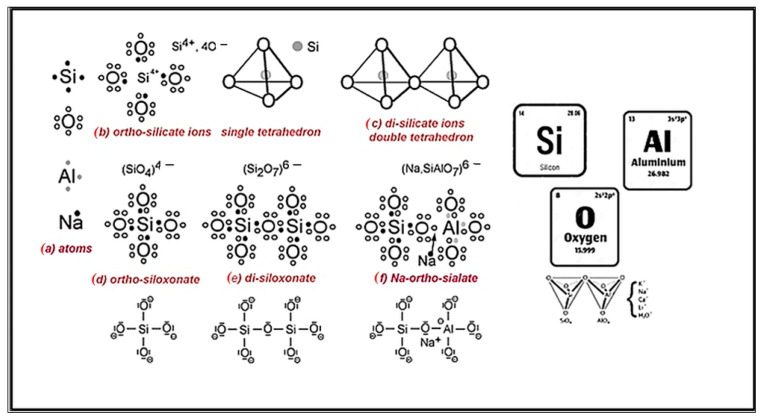
The bonding scheme of geopolymer materials.

Geopolymers are inorganic and amorphous aluminosilicate polymers that constitute a viable alternative to products typically manufactured from Portland cement. The properties of geopolymer materials are primarily dependent on the type of base material (predominantly waste from the energy or mining industries) and the alkali activator utilized [1,2,3]. The formation of geopolymers occurs through the process of polycondensation, wherein [SiO_4_]^4−^ and [AlO_4_]^5−^ tetrahedra interconnect at their vertices, resulting in the development of amorphous or subcrystalline three-dimensional aluminosilicate structures, which exhibit structural similarities to zeolites [4,5]. This process is shown in Figure 1.

The polycondensation process is contingent upon annealing time and temperature. These two factors indirectly influence the final properties of the resultant geopolymer structures [6,7]. These materials exhibit comparable properties to commercially available products. However, with regard to climate protection and environmental impact, they demonstrate significantly enhanced environmental sustainability and energy efficiency. Research has indicated that the production of geopolymers results in CO_2_ and other greenhouse gas emissions up to 10 times lower, and electricity consumption 4–8 times lower than in traditional building materials utilizing Portland cement. Furthermore, these materials possess a substantially reduced carbon footprint and align with the principles of a circular economy [8,9,10]. These materials can be synthesized in solid form (as an alternative to concrete) and in foamed form, referred to as geopolymer foams (serving as an alternative to polystyrene or mineral wool, for instance).

In recent years, increasing attention has been directed toward sustainable construction, which emphasizes the environmental impact of products and materials. It is imperative to not only reduce carbon dioxide emissions but also to manage natural resources judiciously. Consequently, there is a growing demand for materials with superior thermal insulation properties and low density. By 2020, the concentration of carbon dioxide in the atmosphere had increased to 48 percent above pre-industrial levels (by 1750) [11,12,13]. The elevation in the concentration of this gas is attributed to anthropogenic activities, primarily through deforestation and the combustion of fossil fuels [14,15,16,17]. Other greenhouse gases are emitted in smaller quantities as a result of anthropogenic activities. Methane exhibits a higher greenhouse potential than CO_2_ but possesses a shorter atmospheric residence time. Nitrous oxide, similar to CO_2_, is a persistent greenhouse gas that accumulates in the atmosphere for decades or centuries. Non-greenhouse gas pollutants, including aerosols such as particulate matter, demonstrate diverse warming and cooling effects and have been associated with additional environmental concerns, including diminished air quality [18,19,20,21].

The operation of buildings accounts for 30% of global final energy consumption and 26% of energy-related emissions worldwide (8% direct emissions from buildings and 18% indirect emissions from electricity and heat production for buildings). Consequently, the construction industry is expected to play a significant role in ameliorating the current state of environmental pollution and mitigating or reducing emissions of harmful gases. According to the International Energy Agency (IEA) report, the intensity of direct CO_2_ emissions from cement production has remained constant over the past five years, with a slight increase (1%) in 2022. However, to achieve a net zero emissions scenario by 2050 (NZE), it is imperative to attain an annual decrease in CO_2_ intensity of 4% by 2030 for the sector. Strategies to achieve this goal include reducing the clinker-to-cement ratio through the utilization of clinker substitutes, continuously enhancing energy efficiency, adopting low-carbon fuels, improving material efficiency, and implementing innovative technologies such as carbon capture and storage (CCS) [22].

A material that can potentially ameliorate the situation is geopolymer foam materials, the development of which has been notably robust and dynamic in recent years. Geopolymer foams are characterized by a thermal conductivity of less than 0.1 W/m × K (typically 0.06–0.07 W/m × K) and low or very low density. The insulating effect of a foamed geopolymer material is enhanced as its density decreases, thus also reducing its thermal conductivity [23,24,25]. Various substances and components can be incorporated into this type of material, resulting in alterations to its thermal or mechanical properties. In recent years, due to the implementation of new energy management regulations, phase-change materials, which have been known for several decades, have gained increased attention and utilization [26]. The incorporation of phase-change materials frequently compromises the strength properties of composites, necessitating the use of reinforcing additives. Natural fibers represent a promising material in this regard, as they enhance other parameters concurrently. The combination of renewable/natural fibers with PCMs presents an advantageous solution. Numerous researchers recognize the potential in utilizing biomass-based materials in conjunction with PCMs. The authors of [27] elucidated the concept and effects of research concerning the combination of biomass and phase-change material (PCM) components, such as polyethylene glycols, paraffin, and fatty acids. The study concludes that it is essential to exploit the potential properties of biomass and to identify the optimal combination of such materials with those capable of energy storage to achieve novel outcomes. Although the utilization of biomass and its derivative materials in composite PCMs for energy storage has yielded certain advancements for the authors of the study, it remains necessary to further expand the investigation of these raw materials, as the synergistic effects of biomass materials and PCMs require comprehensive examination.

The research conducted by other authors also examines the utilization of PCM in composites reinforced with fibers of non-plant origin. A particularly noteworthy approach involves the incorporation of activated carbon fibers into the composite structure [28]. This material exhibits high thermal conductivity and can be utilized in temperature management systems for lithium-ion batteries. The addition of 1% activated carbon fibers can enhance the thermal conductivity of the composite by up to 130%. Furthermore, investigations have been conducted on the use of basalt and polypropylene fibers in geopolymer composites with PCM [29]. The incorporation of PCM into geopolymer composites resulted in a decrease in strength properties; however, the addition of fibers contributed to an improvement in these properties. Composites utilizing basalt fibers exhibited a higher thermal conductivity coefficient compared to those containing polypropylene fibers.

The utilization of natural fibers in geopolymer composites has been extensively investigated and documented in the scientific literature. Researchers concur that natural fibers, including bamboo, flax, hemp, and jute, are suitable for reinforcing geopolymer matrices, offering additional advantages such as enhanced tensile and flexural strength, reduced density, and improved thermal and acoustic insulation properties. Scholars in this field emphasize that certain characteristics of fiber-reinforced geopolymers, such as the mechanical, thermal, acoustic, hydrophobic, and fire-retardant properties of geopolymer composites, are significantly influenced by the stiffness of the natural fiber, its content, size, and adhesion at the fiber/resin interface. Furthermore, untreated natural fibers are susceptible to fiber pullout and separation, which diminishes their properties. It is therefore recommended that appropriate fiber treatments be implemented, as this can be a crucial step in enhancing fiber/matrix interfacial adhesion, thereby improving mechanical, thermal, and water absorption properties in natural fiber-reinforced geopolymer composites [30,31]. Results pertaining to the utilization of flax and hemp fibers in geopolymer composites were also presented in papers [32,33]. While the utilization of natural fibers or biomass as carriers for energy storage materials has been acknowledged, there remains a dearth of comprehensive information regarding the synergistic effects of such solutions. Furthermore, there is a lack of comparative data on the properties of composites incorporating natural fibers versus those containing phase-change materials (PCMs), particularly in terms of their efficacy in energy conservation and thermal insulation applications.

### 1.2. Materials Able to Store Energy

**Figure 2:**
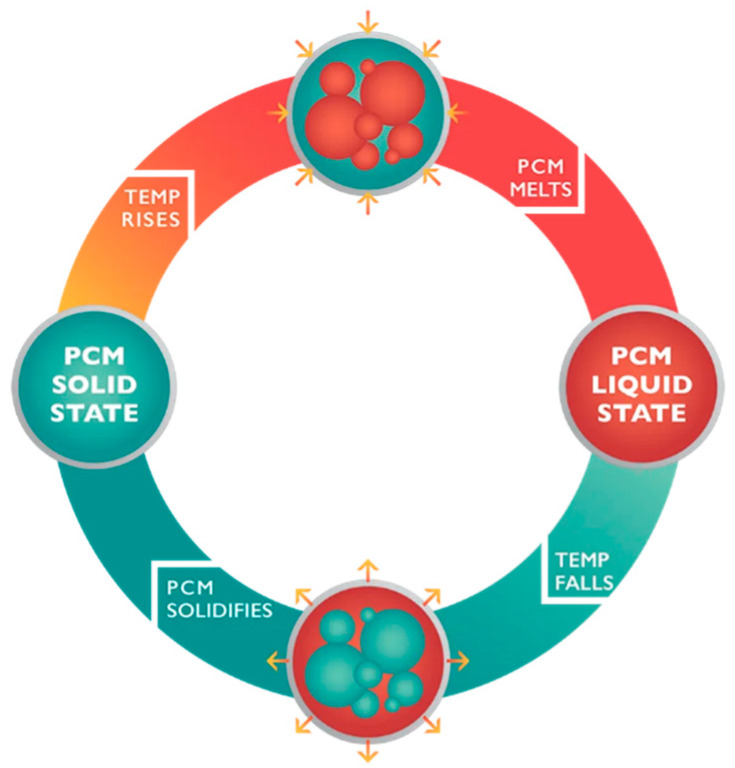
The essence of phase-change materials (permission from Microtek Laboratories Inc.).

Thermal energy storage (TES) materials utilize latent heat. Their distinctive property enables heat storage, and their operation is predicated on exploiting the heat of phase transitions in various systems, such as liquid–solid transitions. This group of materials is generally classified as phase-change materials (PCMs). As the phase-change process is, depending on the direction of the transformation, an exothermic or endothermic process, these materials can be effectively employed for energy accumulation and dissipation [34,35]. Figure 2 illustrates a schematic representation of the operational mechanisms of these materials [36].

Phase-change materials (PCMs) can be classified as organic, inorganic, or eutectic mixtures. Organic PCMs include paraffin and fatty acids. Materials of organic origin exhibit a wide range of melting points. One of the most critical parameters of phase-change materials is thermal conductivity, which facilitates rapid response to changing temperature conditions and ensures operation with minimal temperature fluctuations. The advantages of organic phase-change materials include stability over multiple melt-solidification cycles and solidification without supercooling. Furthermore, they are chemically inert, compatible with engineering materials, and recyclable. Inorganic PCMs comprise eutectics and salt hydrates. These materials, similar to organic PCMs, demonstrate a wide range of melting points. A significant disadvantage of these substances is their corrosive nature. PCMs of inorganic origin are also characterized by instability in transformation cycles, supercooling upon solidification, substantial volume changes during phase transformations, and segregation [37,38]. This study utilizes two distinct phase-change materials of organic origin belonging to the paraffin group.

The utilization of phase-change materials represents a technique that facilitates energy storage, particularly from solar sources, among other applications. These materials find widespread use across various industrial and economic sectors. Potential frameworks for the application of these materials can be identified as follows [39,40,41,42]:

- Thermal energy storage in the structural components of buildings,
- Storage of thermal energy acquired from solar panels or alternative renewable sources,
- “peak shaving”—mitigating fluctuations in electricity demand profiles by reducing peak loads for industrial and commercial electricity consumers,
- Food industry—technological processes with short-term energy requirements, storage of food products,
- The transportation of medical substances, fluids, and organs,
- The temperature stabilization of environments housing electronic equipment,
- Cooling systems for internal combustion engines and electric motors.

At present, numerous companies worldwide are engaged in the production of finished products and the manufacturing of phase-change materials. Among the most prominent entities in this field are PCM Thermal Solutions (Naperville, IL, USA), Environmental Process Systems (Peterborough, UK), Rubitherm (Berlin, Germany), BASF (Ludwigshafen, Germany), THERMOFIN Cristopia Thermal Energy Storage (Montreal, QC, Canada), Climator Sweden AB (Skövde, Sweden), Mitsubishi Chemical Group (Tokyo, Japan), PCM (Dongguan, China) and PCP Australia (Perth, Australia) [43,44,45,46,47,48,49,50]. This paper will present the results of testing geopolymer foams using phase-change materials (PCMs) from Rubitherm. Rubitherm GmbH is a leading supplier of PCMs globally. Their products are utilized in various applications, including air conditioning and heating, temperature regulation, the transportation of specialized goods, and medicine. Rubitherm’s product range includes PX powder and GR granules, which are trade names for their specific formulations. The organic component in the PX material constitutes approximately 60% of its mass. The particle size of the inorganic carrier matrix is 200 µm. This material combination forms a white powder suitable for pouring applications. The GR material has been developed to facilitate ease of pouring. It comprises approximately 30% by weight of organic material. While structurally similar to the porous nature of PX material, the GR formulation differs in its PCM ratio and particle size. The diameter of its granulate structure ranges from approximately 1–3 mm. The characteristics of these products are presented in Table 1 [51,52].

### 1.3. Fibers of Natural Origin

**Figure 3:**
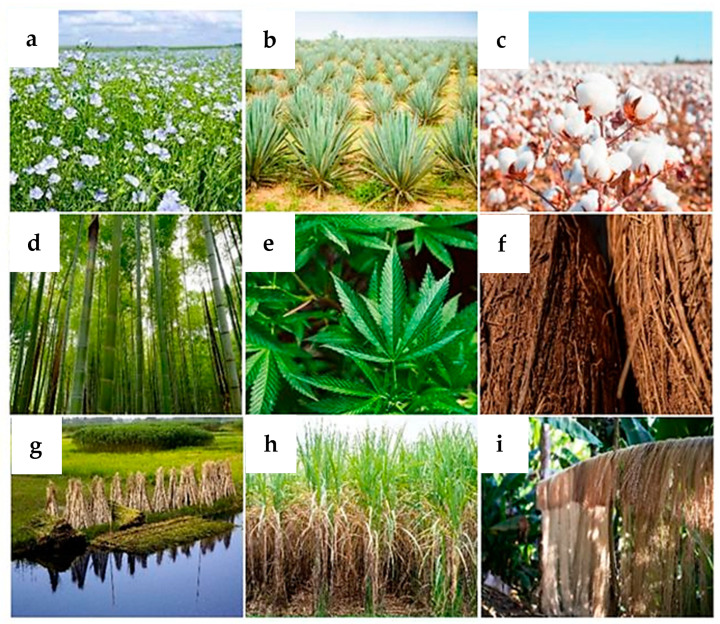
Fibers of natural origin: (**a**) flax, (**b**) sisal, (**c**) cotton, (**d**) bamboo, (**e**) hemp, (**f**) coconut, (**g**) jute, (**h**) sugar cane, (**i**) banana.

Various types of components in the form of fibers of natural origin can be incorporated into geopolymer foams. Natural fibers are categorized into those of plant, animal, and mineral origin. Plant fibers are derived from various parts of plants—seeds, stems, leaves, or fruits. Fibers of animal origin are obtained from mammalian hair and animal secretions. Mineral fibers are fibrous inorganic substances of natural or artificial origin. This study utilizes natural fibers of plant origin; therefore, their characteristics will be discussed subsequently. The most frequently utilized natural fibers of plant origin include flax, sisal, cotton, bamboo, hemp, coconut fiber, jute, sugar cane, and banana (Figure 3) [53,54,55,56].

The primary constituents of cellulose fibers or filaments are cellulose, hemicellulose, and lignin (Table 2). The proportions of cellulose, hemicellulose, and lignin in plants vary due to factors such as maturity, geographical location, environmental conditions, and species. Cellulose is a linear polymer of plant origin, an unbranched polysaccharide that constitutes a component of dietary fiber, among other functions. The molecular formula of cellulose is (C_6_H_10_O_5_)_n_. All plant fibers exhibit a crystalline structure comprising 65–70% cellulose. Furthermore, the properties and characteristics of cellulosic fibers are influenced and modified by lignins and other non-cellulosic components. Owing to the presence of numerous hydroxyl groups and their hygroscopic nature, one of the most significant properties of plant fibers is their enhanced capacity to absorb moisture, which is frequently a crucial requirement for manufactured composites [57,58,59,60,61].

### 1.4. The Essence of the Research Conducted

This research aimed to develop alkali-activated foamed composites incorporating phase-change materials and natural plant fibers, and to compare the thermal and strength properties of the resulting geopolymer structures. Phase-change materials represent an innovative solution in the construction sector, offering significant energy savings, while natural plant fibers serve as a viable alternative to previously utilized mineral fibers. The base material for the geopolymer foam consisted of anthropogenic waste from the energy industry (F-grade fly ash from the Skawina Power Plant). The study also utilized organic phase-change materials from the paraffin group, sourced from Rubitherm, Germany. Hay and wood shavings (from local suppliers) and coconut fibers from Sri Lanka were employed as natural fibers. The incorporation of natural fibers in fly ash-based geopolymer foams represents a novel approach in materials engineering, as such fibers were previously utilized primarily for reinforcing solid composites. The resulting geopolymer exhibits enhanced energy efficiency and environmental sustainability compared to traditional insulation materials. The development of modern materials based on geopolymers, phase-change materials, and natural fibers offers potential for industrial-scale production at reduced costs, while the utilization of waste and renewable fibers mitigates excessive natural resource consumption. Innovative materials with reduced carbon footprints and improved insulation performance can contribute to significant financial savings and environmental benefits associated with reduced energy demand. Furthermore, the incorporation of PCMs in geopolymers represents an innovative approach, as phase-change materials have predominantly been utilized in solid composites and infrequently in foamed materials.

Phase-change materials incorporated into a matrix of porous insulating geopolymer facilitate the additional retention of heat passing through the building envelope by the PCM. Upon the cooling of the partition, this heat is released; however, due to the insulating properties of the partition, it does not fully enter the room, as its entirety is impeded by the porous structure of the matrix material. This comprehensive and thermally efficient solution ensures thermal comfort in the rooms where it is implemented. A building envelope constructed with such a solution will mitigate the risk of room and building overheating. The solutions and research findings described in this article represent an innovative approach to the utilization of PCM in building materials and may contribute significantly to the advancement of disciplines such as civil engineering and materials engineering. Results demonstrating the concept of incorporating PCMs into foamed geopolymers were also presented in works such as [62], where increases of 105% and 181% in heat storage capacity were achieved. Additionally, this topic was partially addressed in the work of [63], wherein PCMs were incorporated into polyurethane foams and subsequently introduced into a geopolymer matrix to create a composite utilized as a thermal insulator.

In conclusion, the incorporation of phase-change materials (PCMs) and natural fibers into foamed lightweight insulating geopolymer materials serves to enhance thermal insulation properties and mechanical characteristics.

1. Phase-change materials (PCMs):
  - Facilitate the storage and release of thermal energy during phase transitions.
  - These materials enhance the thermal insulation properties of materials, which is particularly advantageous in temperature regulation applications.
  - These systems facilitate the maintenance of stable temperatures within buildings, thereby contributing to energy conservation.
2. Natural fibers:
  - Enhance the mechanical properties of materials, including flexural and tensile strength.
  - These materials can enhance the durability and structural stability of foamed insulation materials.
  - Natural fibers exhibit the potential to enhance the antibacterial, antifungal, and anti-allergenic properties of materials.
  - They can also help improve the insulating properties and thermal energy storage capacity, and improve thermal stability [64,65].

The incorporation of PCM and natural fibers into geopolymer materials facilitates the development of more efficient and functional insulation solutions that are environmentally sustainable, energy-efficient, and resistant to diverse thermal conditions. This article presents a comparative study on the utilization of PCM and natural fibers in foamed geopolymer composites.

## 2. Materials and Methods

The geopolymer foams were synthesized utilizing fly ash obtained from the Skawina Power Plant (Skawina, Poland). Fly ash suitable for concrete applications was employed (certificate no: 1488-CPR-0166/W, loss on ignition: category A and B, fineness: category N, declared value 20%, grain density: declared value 2210 kg/m^3^). This fly ash is characterized by a high concentration of silicon dioxide (SiO_2_) and a content of aluminum trioxide (Al_2_O_3_) exceeding 50%. X-ray fluorescence (XRF) oxide analysis was conducted using a SCHIMADZ EDX-7200 instrument (Shimadzu, Kyoto, Japan), and the results are presented in Table 3. These findings are derived from the authors’ previous research.

A cement with the trade name Górkal 70 (Górka Cement, Trzebinia, Poland) was utilized as a hydraulic additive to stabilize the porous structure. The surfactant employed was Syringaldehyde (Merck, Darmstadt, Germany). Sand from the Świętochłowice Sand Plant and ash microspheres, responsible for the presence of closed pores in the composite (TERMO-REX S.A., Jaworzno, Poland), were also incorporated into the mixture. A 10-mole solution of sodium hydroxide with sodium glass (an aqueous solution of sodium silicate) served as the activator of the polycondensation reaction of the geopolymer, and 36% hydrogen peroxide H_2_O_2_ (Grupa Azoty, Puławy, Poland) was utilized to foam the dense-plastic geopolymer mass. Natural plant fibers, specifically hay (Dach-Wkręt, Warsaw, Poland), wood shavings (Dach-Wkręt, Warsaw, Poland), and coconut fiber (producer—PROMAT, Colombo, Sri-Lanka (fibers from Sri-Lanka)), as well as two types of paraffinic phase-change materials, GR42 and PX25 (Rubitherm, Germany), were additionally incorporated into the geopolymer materials. The specifications of natural fibers are presented in Table 4, while those of phase-change materials are presented in Table 5.

**Figure 4:**
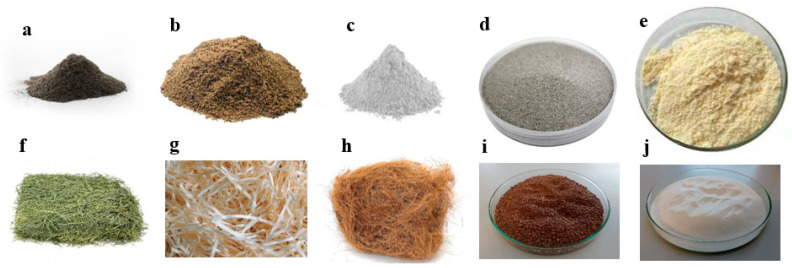
Materials used for the fabrication of the samples (**a**–**e**) and additives (**f**–**j**): (**a**) fly ash, (**b**) sand, (**c**) cement, (**d**) fly ash microspheres, (**e**) Syringaldehyde, (**f**) hay, (**g**) wood shavings, (**h**) coconut fibers, (**i**) GR42, (**j**) PX25.

Following the evaluation of materials utilized in the formation of geopolymer foams, finalized samples were produced and prepared. Figure 4 illustrates all materials employed in the experiments, while Table 6 presents the specimen designations and specifies the quantities of components used in foam production. Table 7 delineates the final dry composition of the products, including the mass and percentage values of each constituent. “R.F.A.” denotes the reference fly ash sample. Consistent quantities of surfactant, cement, sand, and microspheres were utilized in the preparation of all sample variants. Additionally, equivalent amounts of the primary precursor fly ash and foaming agent were employed across all samples.

### Preparations of Geopolymer Foams

**Figure 5:**
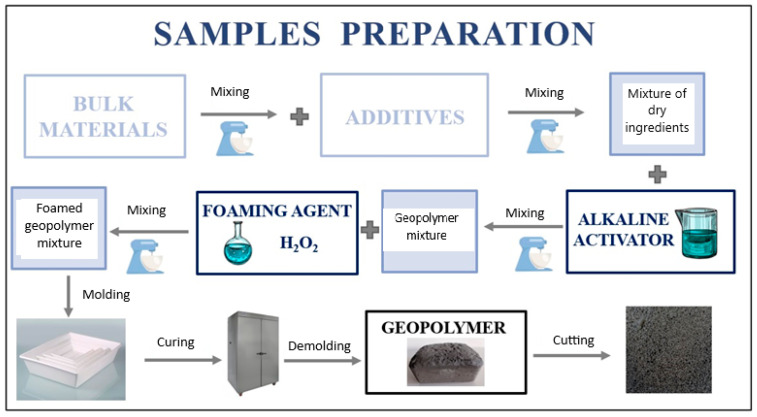
Diagram of geopolymer foams manufacturing. Indication of the various processes from multi-step mixing of ingredients to curing and sample molding.

All the bulk ingredients, i.e., fly ash, sand, cement, ash microspheres, PX25 and GR42 phase-change materials, and Syringaldehyde surfactant were combined until the ingredients were homogeneously distributed (for 10 min in a slow mixer according to EN 196 [69]). All natural fibers were subsequently added in the form of long staple fibers approximately 5 cm in length to the bulk ingredient mixture. The fibers were not subjected to any special preparation beforehand; they were merely cut to an equal length upon receipt from the manufacturer. No hydrothermal treatment or acid or alkaline treatment was applied to the fibers. Following the mixing of the geopolymer mixture and additives, an alkaline activator in the form of a 10 M sodium hydroxide soda-glass solution (aqueous sodium silicate solution) was introduced. Upon obtaining a dense-plastic mixture, 36% hydrogen peroxide H_2_O_2_ was added. The mixing process was conducted in an M/LMB-s laboratory mixer for approximately 15 min at 58 rpm. After achieving a homogeneous mass and its subsequent foaming, the material was transferred to appropriate molds and then annealed at 60 °C for 24 h in an SLW 750 laboratory dryer (POL-EKO, Wodzisław Śląski, Poland). After 24 h, the samples were demolded and cut into 20 × 20 cm panels. The thickness of the panels was approximately 2.5 cm. A schematic representation of the geopolymer fabrication is presented in Figure 5. All tests were conducted after 28 days. The samples were conditioned at an ambient temperature with 40–60% relative humidity.

## 3. Results

### 3.1. Density of Ready-Made Samples

The density of the manufactured panels was determined utilizing a geometric method based on the mass and volume of the samples. Each panel was replicated twice, with the volume of the panels remaining constant (20 × 20 × 2.5 cm), while the mass exhibited slight variations between samples. Panel density was calculated twice, and the mean value was derived from the two measurements (the total average value was calculated from eight repetitions/measurements). The dimensions of the samples were measured using a laboratory caliper with a precision of 0.01 mm, and the mass of the samples was determined using a RADWAG PS 200/2000.R2 precision laboratory analytical balance (RADWAG, Radom, Poland) with an accuracy of 0.01 g. Table 8 presents the two density measurement results.

The incorporation of both natural fibers and phase-change additives into geopolymer foams resulted in an increase in density. This phenomenon can be attributed to alterations in the consistency of the geopolymer mixture and modifications to the porous structure formation process. Natural fibers, with densities ranging from 34 to 47 kg/m^3^, contribute to the increased density of the resulting composites. The final density values of the composites were further elevated due to the absorption of alkaline solutions by the fibers. Although the prepared composites utilized comparable quantities of liquid activators, it is probable that during the maturation period, the fiber-containing composites exhibited reduced liquid yield compared to the fiber-free samples, potentially due to a lower evaporation rate. Phase-change materials exerted the most significant influence on density increase, with PX25 and GR42 having densities of 650 kg/m^3^ and 800 kg/m^3^, respectively. Among natural fibers, wood shavings at 1 wt.% demonstrated the most substantial density increase (31%). Hay fibers and coir fibers exhibited density increases of 19% and 15%, respectively. Regarding phase-change additives, density increases were observed as follows: 2% for 2.5 wt.% GR42, 30% for 2.5 wt.% PX25, 18% for 7.5 wt.% GR42, and 14% for 7.5 wt.% PX25. The sample containing 2.5% PX25 presented as an outlier among the phase-change materials. These compositions exhibited slight sagging during manufacture, rendering the density increase non-indicative of the quantity of additive introduced. The reduced stability of foamed structures was observed for this particular composition.

### 3.2. Compressive Strength of Ready-Made Samples

**Figure 6:**
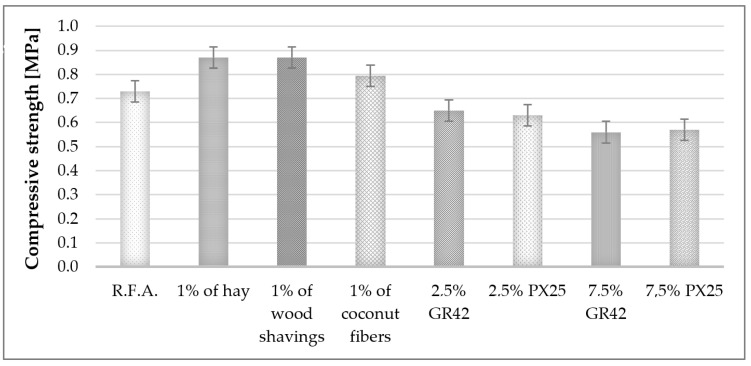
Compressive strength of samples with the addition of natural fibers and phase-change materials. Confirmation of the positive effect of fiber addition on compressive strength.

Compressive strength tests were conducted utilizing an MTS Criterion 43 testing machine (MTS Systems SAS, Créteil, France) equipped with TestSuites 1.0 software, featuring a measurement range of up to 30 kN. The tests were performed to verify the mechanical strength of the specimens. The test speed was maintained at 10 mm/min. The protocols for determining the compressive strength of concrete specimens are delineated in PN-EN 12390-3:2019-07 (Testing of concrete—Part 3: Compressive strength of test specimens) [70]. Additionally, the shape and dimensions of the test pieces are regulated by PN-EN 12390-1:2013-03 [71]. The validity of the test can be ascertained by examining the type of damage observed [72,73]. Ten specimens of each type, with dimensions of 25 × 25 × 25 mm, were prepared for testing. Based on the results presented in Figure 6, a graph was generated to illustrate the effect of the introduced additives on the compressive strength in comparison to the reference specimen without additives.

To determine the mechanical properties of geopolymer foams, compressive strength tests were conducted. The results indicated that natural fibers enhanced the mechanical strength, whereas paraffinic phase-change materials (PCMs) diminished this property. The addition of 1 wt.% hay and 1 wt.% wood shavings increased the compressive strength by 29%, while 1 wt.% coconut fibers resulted in a 14% increase. The incorporation of 2.5 wt.% GR42 did not alter the compressive strength, and the other PCMs reduced it by 14%. These findings were corroborated by the microstructure analysis. Samples containing PCMs exhibited notably larger pores, which consequently reduced the strength properties.

### 3.3. Thermal Parameters of Ready-Made Samples

**Figure 7:**
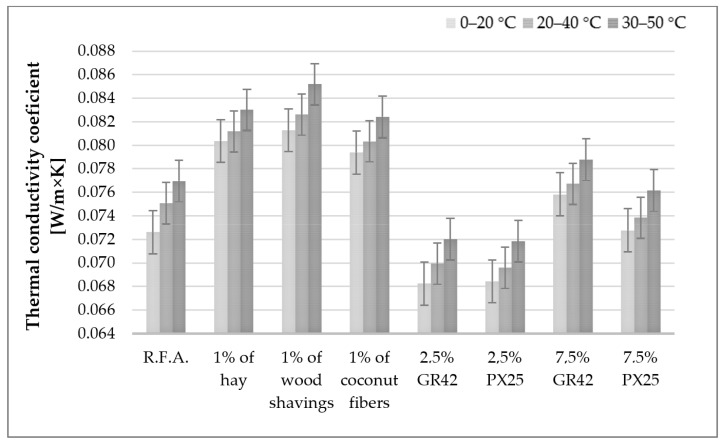
Thermal conductivity of samples with the addition of natural fibers and phase-change materials.

The measurements of thermal conductivity and specific heat were conducted utilizing an HFM 446 plate apparatus (Netzsch, Wunsiedel, Germany). The instrument operates in accordance with established standards, including ASTM C1784 [74], ASTM C518 [75], ISO 8301 [76], EN 12664 [77], and others. The conductivity range extends from 0.007 to 2.0 W/m × K, with a measurement accuracy of ±1–2%, a repeatability of ±0.25%, and a reproducibility of ±0.5%. Temperature regulation and control are facilitated by a Peltier system. The thermal properties of the fabricated foams were determined using the aforementioned device based on the hot and cold panel method. Thermal conductivity was evaluated across three temperature ranges: 0–20 °C, 20–40 °C, and 30–50 °C, while specific heat was assessed within the range of 27.5–32.5 °C. The three temperature ranges for thermal conductivity measurement are presented to elucidate insulation and performance properties. Under actual conditions, insulating materials may operate at temperatures exceeding 30 °C, hence the utilization of three distinct temperature ranges. Figure 7 illustrates the relationship between the thermal conductivity coefficient of the fabricated panels and the measurement temperature.

**Figure 8:**
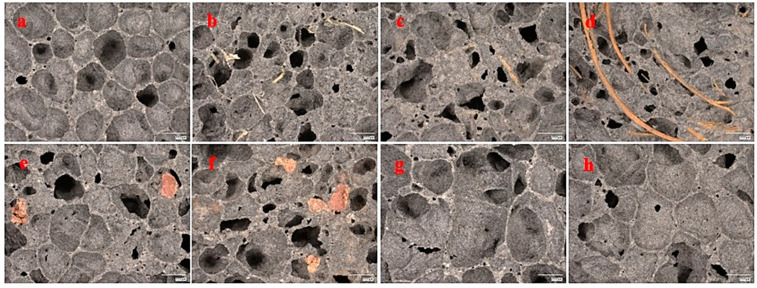
Porous structure morphology: (**a**) reference fly ash, (**b**) 1% of hay, (**c**) 1% of wood shavings, (**d**) 1% of coconut fibers (**e**) 2.5% GR42 (**f**) 7.5% GR42 (**g**) 2.5% PX25 (**h**) 7.5% PX25.

The coefficient of thermal conductivity increases with rising test temperature, which is a well-established physical phenomenon. As the test temperature elevates, the rate of heat transfer mechanism accelerates. All additives in the form of natural fibers increased the coefficient of thermal conductivity, thereby diminishing the insulation performance. Although fibers are utilized as insulating materials, their addition in dispersed small quantities results in an increase in the thermal conductivity coefficient, consequently deteriorating the insulating properties. The lambda coefficient deteriorated by up to 12% for 1 wt.% wood shavings. Materials modified with GR42 and PX25 additives at 2.5 wt.% exhibited the lowest value of the thermal conductivity coefficient. The decrease was 6%. For higher contents of these modifiers, 7.5 wt.% slightly increased the lambda coefficient, while 7.5 wt.% PX25 produced no change. The addition of paraffin-based phase-change materials with high thermal conductivity was expected to increase the thermal conductivity coefficients of composites containing them. However, studies demonstrate that these coefficients not only did not increase but even decreased. This phenomenon is attributed to the formation of larger pores in these composites, as evidenced in Figure 8. The marker in the figures corresponds to 2000 μm.

Table 9 presents the results of the specific heat measurements for all geopolymer foams. Two tests were conducted for each variant of the finished samples, which are included in the table and display the mean value and standard deviation. The composite’s heat capacity was calculated based on the specific heat and density.

The analysis of the results of specific heat measurements revealed that the specific heat of all tested samples is at a low level. The differences between the various foam variants are minimal. This effect is evident due to the introduction of a very small amount of additives into the geopolymer matrix, specifically 1 or 2.5 wt.%. Only the addition of 7.5 wt.% GR42 increased the specific heat by approximately 14%. When converting these values to volumetric heat capacity, higher values were obtained for the foamed composites with additives compared to the reference sample. The highest volumetric heat capacity values were observed for materials with 1 wt.% wood shavings additive and 7.5 wt.% GR42 additive (approximately 35%). The interpretation of these results is challenging in terms of determining specific relationships, as the interactions of multiple factors are evident, such as the effect of the additives on the mixture’s consistency and its degree of foaming, the density of the obtained composites, and the heat capacities of the introduced additives. Even the addition of larger amounts of PCMs with a high heat capacity can result in a lower heat capacity of the entire composite, as the introduced PCM additive may affect the consistency of the mixture, resulting in a material with different porosity. This issue necessitates comprehensive research and analysis.

### 3.4. Visual Porosity Assessment of Ready-Made Samples

The image analysis of the fabricated foams was conducted utilizing a Keyence VHX-7000 digital optical microscope (KEYENCE INTERNATIONAL, Mechelen, Belgium). Micrographs of the porous structures of the material are presented in Figure 8. A magnification of 20× was employed. The scale bar in the figures represents 2000 μm.

An analysis of the structure through digital optical microscopy facilitated the determination of pore size and their qualitative distribution. All images reveal the macropores of the produced structures, which are predominantly closed pores. It was observed that the reference ash sample exhibited medium-sized pores with high homogeneity. In the reference ash sample, pores with dimensions ranging from 1500 to 3000 μm were observed. All samples containing natural fibers demonstrated significant pore heterogeneity. The fiber components marginally disrupted the porous structure. For these samples, the pore size primarily ranged from 750 to 2000 μm. Samples incorporating phase-change materials (second row) exhibited high pore heterogeneity for the GR42 additive and the high homogeneity of pores, shapes, and sizes for samples with the PX25 additive. The pore size for samples containing the GR42 additive ranged from 1000 to 2500 μm. The pore size for samples with the PX25 additive ranged from 2000 to 4000 μm.

### 3.5. Microstructure of Ready-Made Samples

**Figure 9:**
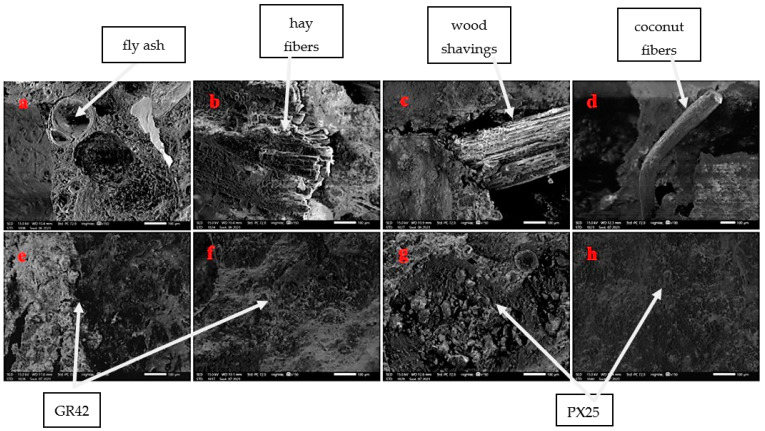
Microstructure of the sample: (**a**) reference fly ash, (**b**) 1% of hay, (**c**) 1% of wood shavings, (**d**) 1% of coconut fibers (**e**) 2.5% GR42 (**f**) 7.5% GR42 (**g**) 2.5% PX25 (**h**) 7.5% PX25.

A JEOL IT200 scanning electron microscope (SEM) (JEOL, Akishima, Tokyo, Japan). was utilized to capture images of the microstructure of the fabricated geopolymer foams. Figure 9 illustrates the morphology of geopolymer structures incorporating natural fibers and phase-change materials. The micrographs were obtained at a magnification of 150×. The scale bar in the figures represents 100 μm.

The SEM image above illustrates the amorphous structure of pure geopolymer and samples with natural fibers and phase-change materials incorporated. Arrows indicate undissolved ash particles, natural fibers, and paraffin additives, which are discernible at high magnification. Natural fibers exhibit poor adhesion to the geopolymer matrix, whereas paraffin demonstrates superior bonding, as evidenced in the figure presented. In Figure 9b, hay fibers that have undergone delamination due to alkali exposure are observed, while in Figure 9c (wood), individual wood fibers are visible within the structure. The coir fiber depicted in Figure 9d displays weak adhesion to the matrix. The absence of geopolymer matrix on its surface may indicate the poor adhesion of this fiber to the geopolymer. Phase-change materials (PCMs) are faintly visible in the geopolymer matrix, suggesting a homogeneous mixture, an absence of PCM particle agglomeration, and a high degree of bonding between the particles and the matrix.

## 4. Discussion

The studies conducted in this publication demonstrated that each material property is dependent on the type of component introduced (in the case of natural fibers of plant origin) and the mass fraction of phase-change materials. This investigation analyzed physical, mechanical, and thermal properties, and performed qualitative image analysis utilizing optical microscopy and scanning electron microscopy.

The highest density among foams incorporating natural fibers was observed in the sample containing wood shavings (approximately 300 kg/m^3^), while other samples exhibited densities of 270 kg/m^3^. The results indicated an inverse relationship between the density of raw fibers and the final density of the produced composite. Wood shavings exhibited the lowest density as pure natural fiber, whereas coconut fiber demonstrated the highest density. Foams containing coconut fibers displayed the lowest density among samples with natural fibers, measuring 263.37 kg/m^3^. Regarding the density of foams incorporating phase-change materials (PCMs), samples with 2.5 wt.% PCM content yielded densities of approximately 230 kg/m^3^ for GR42 and 295.30 kg/m^3^ for PX25. The foam containing 2.5 wt.% PX25 experienced sinking during manufacture, resulting in an excessively high density relative to the amount of modifier introduced. The 7.5 wt.% PCM content resulted in foams with this additive exhibiting a density of approximately 260 kg/m^3^. The density of the reference ash sample was 228.39 kg/m^3^, suggesting that each additional component increased the composite’s density. Other researchers investigated the density of foamed geopolymer composites incorporating cotton flocks. The density of their reference ash sample was significantly higher than that of the reference sample in the present study, measuring 390 kg/m^3^. The authors of this article also examined the density of samples containing 0.5, 1, and 2 wt.% cotton flock, observing a decrease in density with increasing additive content. The optimal results were obtained for 2 wt.% cotton fiber, yielding a density of 350 kg/m^3^ [78]. The results obtained are significantly higher compared to the samples presented in this article. This represents the sole example of foamed geopolymer composites based on fly ash with the addition of natural fibers. To date, other researchers have not produced ash-based geopolymer foams with the addition of natural fibers, thus constituting a novel contribution to the field of materials engineering. While numerous references exist regarding geopolymer concretes with natural fibers, foams remain a niche and relatively unexplored topic. A recent review article [30] indicates that this area has yet to be thoroughly investigated. Although mentions of foamed geopolymer foams exist in this work, the papers demonstrate minimal foaming agent content, and the samples more closely resemble solid materials. Only the research conducted by [79] presents diatomite foams foamed with silicon powder incorporating a vegetable surfactant and hemp fibers; however, the density of these foams is considerably higher (approximately 360 kg/m^3^), and more importantly, they are not fly-ash-based foams, precluding direct comparison. Regarding the density of foams with the addition of phase-change materials, the author’s previous work [66], despite utilizing different modifier contents, yielded similar density results (approximately 200–300 kg/m^3^). Other researchers [80] examined the density of insulating panels for interior wall panels with the organic phase-change composite MikroCaps (MikroCaps, Ljubljana, Slovenia). Their findings revealed that the density of the produced composite was 290 kg/m^3^, aligning with both the present and earlier work of the authors.

An analysis of the mechanical properties, specifically compressive strength, demonstrated that natural fibers enhanced mechanical strength (from 0.7 MPa to 0.8 or 0.9 MPa), whereas paraffinic PCMs diminished this property (to 0.6 MPa). The 1 wt.% hay and 1 wt.% wood shavings increased the compressive strength by 29%, while 1 wt.% coconut fibers increased it by 14%. The 2.5 wt.% GR42 did not alter the compressive strength, and the other PCMs decreased it by 14%. In the study [79], compressive strength results of 1.2–2.8 MPa were obtained with the addition of hemp fibers, but these are diatomite-based foams, not fly-ash-based as in that study. It is only possible to compare these to the work of [78], where results exceeding 1 MPa were obtained, which was not achieved in this study; however, the compressive strength remains sufficient for the utilization of the produced composites as insulating materials. In the authors’ previous work [66], analyzing geopolymer foams with the addition of phase-change materials, it was demonstrated that the addition of PX25 reduced the compressive strength for all modifier contents (5, 10, 15 wt.%). The 5 wt.% of GR42 additive decreased the compressive strength, and the addition of only 10 and 15 wt.% increased the strength parameters of the samples by 14 and 29%, respectively. Thus, the compressive strength of foams increases only when a sufficient amount of modifier is added. In this study, only 2.5 and 7.5 wt.% were used, rendering the strength increase unattainable. Other researchers [81] investigated the effect of PCM on the strength properties of geopolymer mortar and observed that the addition of PCM leads to a minor decrease in compressive strength. This decrease is minimal, and the compressive strength of geopolymer mortar with up to 20% PCM added remains adequate for use in construction.

An analysis of the thermal conductivity coefficient tests revealed that higher test temperatures corresponded to higher thermal conductivity coefficients, which is consistent with established physical principles, as elevated temperatures facilitate more rapid heat transfer. All additives in the form of natural fibers increased the thermal conductivity coefficient, thereby diminishing the insulation performance. The thermal conductivity coefficient deteriorated by up to 12% for 1 wt.% wood shavings. The lowest value of the thermal conductivity coefficient was observed for materials modified with GR42 and PX25 additives at 2.5 wt.%. The decrease was 6%. For higher concentrations of these modifiers, 7.5 wt.% slightly increased the thermal conductivity coefficient, while 7.5 wt.% PX25 produced no change. The increase in thermal conductivity values for fiber composites was an unexpected phenomenon, as natural fibers are frequently utilized as insulating materials. Consequently, a decrease in the value of the thermal conductivity coefficient would have been anticipated. In this instance, the obtained results can be attributed to the use of fibers that had not undergone prior preparation or appropriate treatment. Natural fibers possess high absorbency, and when exposed to liquid activating solutions, the fibers absorbed a portion of the liquid, which altered their heat conduction properties. This observation is corroborated by the density results of the composites obtained. Samples containing natural fibers in each variant exhibited a higher density than the reference sample without the addition of fibers. The increase in the coefficient of thermal conductivity of saturated fibers should be considered as both a favorable and unfavorable phenomenon. On the one hand, if one prioritizes the favorable insulating properties of the composite, the utilization of non-saturated fibers is disadvantageous due to the increase in density and the thermal conductivity coefficient. On the other hand, the employment of reinforcing fibers that additionally contribute to increasing the heat capacity of the composite is most advantageous, and this was the case in the research presented in this article. The study [78] obtained thermal conductivity results of 0.12–0.16 W/m × K (ash-based foams with cotton flock), which is relatively high for these materials. In this publication (foams with hay, wood shavings, and coconut fiber), the thermal conductivity was no higher than 0.086 W/m × K and typically stabilized at a constant level of approximately 0.080 W/m × K. Diatomite foams foamed with silicon powder with vegetable surfactant and hemp fibers [79] yielded thermal conductivity coefficient results ranging from 0.06 to 0.07 W/m × K. In the case of foams with phased materials, this publication obtained results similar to previous work. Previously [66], the lowest value of thermal conductivity coefficient was obtained for materials modified with 5 wt.% of GR42 additive and 10 wt.% of MikroCaps additive. An amount of 15 wt.% of each additive increased the thermal conductivity coefficient. The results indicated that thermal conductivity is most effectively reduced by a low PCM additive; therefore, very small amounts of phase-change modifiers were utilized in this work. Other researchers developing PCM insulation demonstrated that the thermal conductivity of the samples ranged from 0.064 to 0.074 W/m × K. The increase in conductivity is determined by a reduction in the porosity of the insulating material [80,82]. The thermal conductivities for similar unconventional natural raw materials range from 0.06 to 0.1 W/m × K [83,84], which closely aligns with the measured values.

An analysis of the specific heat measurement results indicated that the specific heat of all samples tested is approximately 1 kJ/kg × K. The variations between different foam variants are minimal. This effect is apparent due to the introduction of a very small quantity of additives into the geopolymer matrix, specifically 1 or 2.5 wt.%. Only the addition of 7.5 wt.% GR42 increased the specific heat by nearly 14%. Regarding the measurement of specific heat in foams with the addition of natural fibers, no literature data were found on this subject, which clearly demonstrates the novelty of this field of research. Conversely, several publications describe the issue of foams with phase-change materials. In the authors’ previous work [66], parameters in the range of 1387–1943 kJ/kg × K were obtained during the study of specific heat. For samples with 15 percent PCM, the specific heat was the highest for any phase-change additive. The specific heat of insulating boards measured by other researchers ranged from 1.4 to 1.8 kJ/kg × K. The highest specific heat value was observed for the sample with the incorporated phase-change material, and this value was 28% higher than for materials without PCM. [80,82]. Additional research has demonstrated that increased PCM content correlates positively with improved specific heat results [35,52]. Upon the conversion of specific heat values to volumetric heat capacity, the foamed composites with additives consistently exhibited higher values compared to the reference sample. The most significant volumetric heat capacity values were observed in materials containing the 1 wt.% wood shavings additive and 7.5 wt.% GR42 additive, representing an increase of approximately 35% [85,86].

The conducted research and its results are significant not only from a scientific or cognitive perspective but also in terms of the potential applications of such materials. The comparison of insulating and heat-accumulating capabilities between natural fiber materials and phase-change materials based on paraffin is of considerable importance. The knowledge that natural fibers can affect heat capacity to a similar extent is valuable and may contribute to the increased applications of such materials. Frequently, the primary barrier to the widespread adoption of PCMs is their cost. The scientific community engaged in these areas should prioritize the development of this type of research to facilitate the future development of simple and cost-effective technologies for utilizing natural fibers in construction, improving the insulation parameters of building partitions, and enhancing their thermal energy storage capacity.

## 5. Conclusions

Based on the aforementioned discussion of the research results, several conclusions can be drawn to summarize the research work:

I. The highest density among foams with natural fibers was observed in the sample containing wood shavings (approximately 300 kg/m^3^). The results indicated an inverse relationship between the density of the raw fibers and the final density of the produced composite. Each component incorporated into the geopolymer foams, including both natural fibers and phase-change additives, contributed to an increase in density.
II. Natural fibers enhanced mechanical strength, whereas paraffinic PCMs diminished this property. The addition of 1 wt.% hay and 1 wt.% wood shavings resulted in a 29% increase in compressive strength, while 1 wt.% coconut fibers led to a 14% increase. The incorporation of 2.5 wt.% GR42 did not alter the compressive strength, whereas other PCMs reduced it by 14%.
III. All additives in the form of natural fibers increased the coefficient of thermal conductivity, thereby compromising the insulation performance. The lambda coefficient deteriorated by up to 12% for 1 wt.% wood shavings. The lowest value of the thermal conductivity coefficient was obtained for materials modified with GR42 and PX25 additives at 2.5 wt.%, with a decrease of 6%.
IV. The incorporation of both phase-change materials and natural fibers positively influenced the obtained values of the volumetric heat capacity of the composites. This effect is attributed to the varying densities of foamed composites and the heat capacity of the additives utilized.

Based on the aforementioned test results, it can be concluded that an increase in strength can be achieved by incorporating as little as 1% by weight of natural fibers of plant origin. Modifiers in the form of natural materials (up to 20 wt.%) consistently result in a slight reduction in strength parameters; however, these results remain sufficient in terms of the required insulation standards. The addition of both natural fibers and phase-change materials to geopolymer foams yields favorable results with respect to the thermal conductivity coefficient. A high level of specific heat can be attained only with a higher content of the variable-phase additive (approximately 15 wt.%). The presented research results constitute a valuable contribution to the field of insulating research on fiber-reinforced materials and materials containing variable-phase materials. As the study demonstrated, the addition of only a few percent of PCMs does not result in a significant increase in heat capacity, and this increase can be equally well achieved by incorporating natural fibers into foamed geopolymers. The research corroborated reports by other authors regarding the fact that the addition of fibers to building materials/concrete can adversely affect the insulating properties of such materials and increase the thermal conductivity coefficient. Further research is warranted in this area, as foamed geopolymer materials present an attractive alternative to commonly utilized organic polymer or mineral wool insulation. However, foamed geopolymers exhibit poor strength properties; thus, reinforcing them with fibers may enhance these parameters and increase the feasibility of this technology for implementation. Natural fibers merit particular consideration, as they are low-carbon and renewable materials. The incorporation of PCMs into foamed geopolymers can potentially increase their heat capacity, which is of significant importance for applications in building envelopes. Nevertheless, these materials are not always economically viable at present, necessitating the exploration of alternative methods to increase thermal capacity. In the context of foamed geopolymers for insulation applications, the addition of natural fibers may yield comparable results to the use of phase-change materials. This innovative approach, comparing the efficacy of different materials in terms of energy accumulation in geopolymers, will be further investigated by the authors.

Future research should focus on the mechanisms of heat transfer through partitions composed of geopolymers and fibers. Of particular interest are multilayer partitions, in which the heat transfer coefficient may be reduced not primarily due to the properties of the individual layers themselves, but rather to the formation of additional thermal resistance at the interface between the fiber layers (insulation) and the geopolymer matrix. Furthermore, subsequent investigations should explore alternatives to commercially available PCMs. The authors are currently conducting research related to the development of cost-effective PCMs produced based on paraffin and mineral sorbents for the sorption of petroleum substances.

## Figures and Tables

**Table 1:** Characteristics of PX and GR phase-change materials.

PX	GR
High heat capacity, Heat accumulation and dissipation take place at constant temperatures, Constant volume of material during phase change, Stability over multiple phase transformation cycles, Long life of the material, Chemical inertness, Non-toxicity, Non-corrosivity, Degradability.	High heat capacity, Heat accumulation and dissipation take place at constant temperatures, Constant volume of material during phase change, Stability over multiple phase transformation cycles, Long life of the material, Non-toxicity, Degradability, Particle size diversity.

**Table 2:** Chemical components, physical and mechanical properties of natural materials.

		Composition		Physical Properties			Mechanical Properties		
Fibers	Cellulose (wt.%)	Hemicellulose (wt.%)	Lignin (wt.%)	Moisture Content (wt.%)	Density (g/cm^3^)	Diameter (µm)	Tensile Strength (MPa)	Young’s Modulus (GPa)	Elongation at Break (%)
Bamboo	26–43	30	21–31	11–17	0.9	10–30	250–850	9.8	5.6–8.6
Banana	63–64	17–19	3–5	8–10	1.35	160–200	355	33.8	53
Coir	36–43	0.15–0.25	40–45	8	1.15–1.46	100–460	131–220	4–6	15–40
Cotton	82–90	5.7	-	7.85–8.5	1.5–1.6	12–38	287–800	5.5–12.5	7–8
Flax	71	18.6–20.6	2.2	8–12	1.5	40–600	88–1500	27.6	2.7–3.2
Hemp	70.4–74.4	17.9–22.4	3.7–5.7	6.2–12	1.47	25–500	550–900	70	1.6
Jute	61–71.5	13.6–20.4	12–13	12.5–13.7	1.3–1.49	25–200	393–800	13–26.5	1.16–1.8
Sisal	67–78	10–14.2	8–11	10–22	1.45	50–200	468–700	9.4–22	3–7
Sugar cane	55.2	16.8	25.3	20–28	1.2	320–400	20–290	19.7–27.1	1.1

**Table 3:** Oxide analysis for fly ash [66].

	Oxide Composition (wt.%)							
Precursor	SiO_2_	TiO_2_	Fe_2_O_3_	Al_2_O_3_	CaO	MgO	K_2_O	Na_2_O
Fly ash	55.9	1.09	5.92	23.49	2.72	2.61	3.55	0.59

**Table 4:** Parameters of natural fibers.

Fibers	Density [kg/m^3^]	Thermal Conductivity [W/m × K]
Coconut fibers	47.15	0.04667
Hay	46.35	0.04239
Wood shavings	34.06	0.04424

**Table 5:** Parameters of phase-change materials [67,68].

	GR42	PX25
Melting point [°C]	38–43	22–25
Solidification temperature [°C]	43–37	25–22
Heat capacity [kJ/kg]	55	95
Specific heat [kJ/kg × K]	2	2
Thermal conductivity [W/m × K]	0.2	0.1
PCM content [%]	30	60
Appearance	brown granules	white powder

**Table 6:** Characteristics of ready-made samples.

ID	Fly Ash Mass [kg/m^3^]	Sand Mass [kg/m^3^]	Cement Mass [kg/m^3^]	Microsphere Mass [kg/m^3^]	Surfactant Mass [kg/m^3^]	PCM/Fibers Mass [kg/m^3^]	H_2_O_2_ [kg/m^3^]	10 M Solution [kg/m^3^]
R.F.A.	116.86	11.76	14.70	23.52	0.73	-	7.35	53.65
1% of hay	116.86	11.76	14.70	23.52	0.73	1.76	7.35	51.45
1% of wood shavings	116.86	11.76	14.70	23.52	0.73	1.76	7.35	51.45
1% of coconut fibers	116.86	11.76	14.70	23.52	0.73	1.76	7.35	51.45
2.5% GR42	116.86	11.76	14.70	23.52	0.73	4.41 GR42	7.35	54.39
2.5% PX25	116.86	11.76	14.70	23.52	0.73	4.41 PX25	7.35	51.45
7.5% GR42	116.86	11.76	14.70	23.52	0.73	14.77 GR42	7.35	55.86
7.5% PX25	116.86	11.76	14.70	23.52	0.73	14.77 PX25	7.35	54.39

**Table 7:** Dry composition of products with mass and percentage of each ingredient.

ID	Fly Ash Content [g/wt.%]	Sand Content [g/wt.%]	Cement Content [g/wt.%]	Microsphere Content [g/wt.%]	Surfactant Content [g/wt.%]	PCM/Fibers Content [g/wt.%]	Geopolymer/Component [wt.%]
R.F.A.						0	100/0
1% of hay	795/70	80/7	100/8.6	160/14	5/0.4	12/1	99/1
1% of wood shavings						12/1	99/1
1% of coconut fibers						12/1	99/1
2.5% GR42						30/2.5	97.5/2.5
2.5% PX25						30/2.5	97.5/2.5
7.5% GR42						100.5/7.5	92.5/7.5
7.5% PX25						100.5/7.5	92.5/7.5

**Table 8:** Density of samples with the addition of natural fibers and phase-change materials.

ID	R.F.A.	1% of Hay	1% of Wood Shavings	1% of Coconut Fibers	2.5% GR42	2.5% PX25	7.5% GR42	7.5% PX25
Mean value [kg/m^3^]	228.39	271.96	299.11	263.37	232.81	295.30	268.58	261.18
Standard deviation [kg/m^3^]	3.51	4.07	5.28	5.64	4.50	3.06	6.28	5.06

**Table 9:** Specific heat and heat capacity of samples with added natural fibers and phase-change materials.

ID	R.F.A.	1% of Hay	1% of Wood Shavings	1% of Coconut Fibers	2.5% GR42	2.5% PX25	7.5% GR42	7.5% PX25
Cp [kJ/kg × K]	1.076	1.009	1.104	1.022	1.115	1.011	1.221	1.063
Standard deviation [kJ/kg × K]	0.102	0.018	0.008	0.001	0.106	0.198	0.029	0.023
Cv [kJ/m^3^ × K]	245.74	274.41	330.22	269.16	259.58	298.55	327.94	277.63

## Data Availability

The original contributions presented in the study are included in the article, further inquiries can be directed to the corresponding authors.
